# Research Progress in Boron-Modified Phenolic Resin and Its Composites

**DOI:** 10.3390/polym15173543

**Published:** 2023-08-25

**Authors:** Li Zhang, Xueshu Zhang, Ruidong Wang, Yifei Zhang, Juntao Wu, Zhimao Zhou, Penggang Yin

**Affiliations:** 1 Key Laboratory of Bio-Inspired Smart Interfacial Science and Technology of Ministry of Education, School of Chemistry, Beihang University, Beijing 100191, China; zhang21@buaa.edu.cn (L.Z.); shu0826@163.com (X.Z.); wrd1018@126.com (R.W.); fff12306@126.com (Y.Z.); pgyin@buaa.edu.cn (P.Y.); 2 CAS Key Laboratory of Green Process and Engineering, Institute of Process Engineering, Chinese Academy of Sciences, Beijing 100190, China

**Keywords:** BPF, composite, synthesis, modification, property

## Abstract

As one of the most successful modified phenolic resins, boron-modified phenolic resin (BPF) has excellent heat resistance and ablative resistance, good mechanical and wear resistance, and flame retardancy. BPF and its composites can be widely used in areas such as aerospace, weapons and equipment, automobile brakes, and fire retardants. In this review, the current state of development of BPF and its composites is presented and discussed. After introducing various methods to synthesize BPF, functionalization of BPF is briefly summarized. Particular emphasis is placed on general methods used to fabricate BPF-based composites and the heat resistance, ablative resistance, mechanical property, wear resistance, flame retardancy, and water resistance of BPF-based composites. Finally, the challenges of this research area are summarized and its future outlook is prospected.

## 1. Introduction

Phenolic resin was the earliest synthetic resin to realize industrial production. It is formed by polycondensation of phenols (phenol, resorcinol, etc.) and aldehydes (formaldehyde, furfural, etc.). Due to its low cost, simple process, good ablation resistance, mechanical properties, and dimensional stability, phenolic resin has been widely used in ablation-resistant materials, friction materials, and fire-resistant materials [1]. However, the phenolic hydroxyl groups and methylene groups of phenolic resins are prone to oxidative degradation [2], which limits their large-scale applications in ablation-resistant materials and other fields, for which they need to be modified.

At present, boron-modified phenolic resin (BPF) is one of the most successful modified phenolic resins. Its research began in the United States in the 1950s and was commercialized in the 1960s. In China, BPF was successfully developed and mass produced in the 1970s by Beijing Composite Materials Co., Ltd. (Beijing, China) and Hebei University [3]. Since the bond energy of B-O bonds is higher than that of C-C bonds [4], the heat resistance and ablation resistance of BPF are much higher than those of general phenolic resins. The inorganic boron compounds can promote char formation in the burning process, thus enhancing the flame retardancy and ablation resistance of BPF. At the same time, since the bond length of the B-O bond is longer than that of the C-C bond, BPF has better flexibility and improved mechanical properties than those of general phenolic resins. However, the three functional boron structures in BPF make it difficult to control the synthesis process and the product is brittle, which restricts the large-scale production and application of BPF. This review aims to provide a snapshot of the recent progress in BPF and its composites and to bring attention to this field. The synthesis methods and modification pathways of BPF are summarized. The molding methods and properties of BPF-based composites are illustrated. Finally, challenges and future perspectives are briefly discussed.

## 2. Synthesis Method of BPF

The synthesis methods of BPF can be broadly categorized into three categories: the paraformaldehyde method, salicyl alcohol method, and copolymerization and blending method. The most common modifier used in BPF synthesis is boric acid.

### 2.1. Paraformaldehyde Method

**Figure 1:**
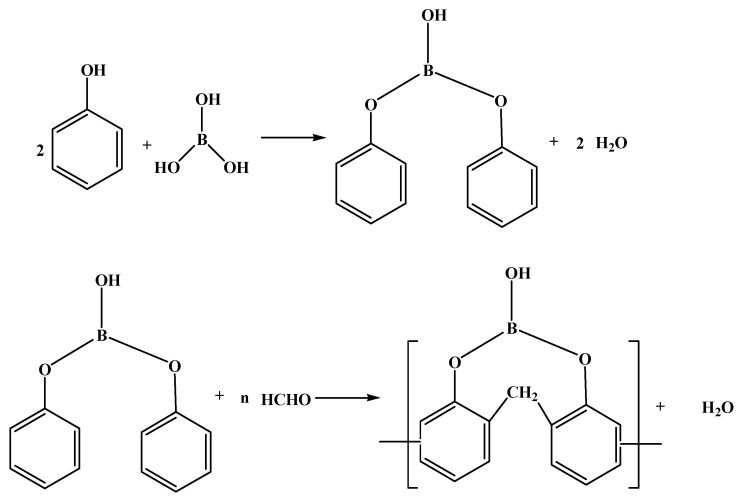
The synthetic route of BPF by paraformaldehyde method [5].

The paraformaldehyde method [5] involves an initial esterification step wherein phenolic compounds like phenol are esterified with boric acid or boron oxide to generate boric acid phenol ester intermediate products. Subsequently, the intermediate products are condensed with paraformaldehyde to prepare BPF (Figure 1).

In the early stages, boron oxide was used as a raw material and the reaction conditions for synthesizing BPF were harsh, with an esterification reaction occurring at 300 °C. These conditions were not suitable for industrial-scale production, and the resulting product exhibited poor processing characteristics [6,7]. Lei Wang et al. [8] utilized boric acid as a raw material and introduced a catalyst, which allowed for a reduction in the esterification reaction temperature to 180 °C. This modification enabled the production of BPF with stable performance in industrial equipment. When the molar ratio of boric acid:phenol:paraformaldehyde is 1:3:3, the comprehensive performance of the product is the best. The heat resistance of the resulting BPF is better than that of the barium phenolic resin and the aminophenolic resin. At 600 °C, the barium phenolic resin completely loses its weight, and the weight loss rate of the aminophenolic resin is as high as 71.5%, while the weight loss rate of the BPF is only 27.7%. In the esterification stage of the reaction, the temperature of the column head should be controlled to be lower than 100 °C to prevent phenol from being carried out and affecting the material ratio of the system, and the feeding temperature in the polycondensation stage should be lower than 40 °C to avoid volatilization of formaldehyde gas. During the experiment, the authors found that the synthesis conditions of this method were harsh and the temperature of the column head was difficult to control.

The reactivity of boric acid can be improved and the esterification reaction temperature can be reduced when toluene is used as a dehydrating agent [9]. The maximum boron content of the synthesized BPF can reach 9.0%, and the residual carbon rate at 1200 °C is as high as 65.3%. The boron content and residual carbon rate of the synthesized BPF were significantly higher than those of commercially available BPF, and the heat resistance and impact toughness increased with the increase in boron content. However, when the boron content was higher than 6.6%, the crosslinking degree of BPF became excessively high, resulting in decreased impact toughness. The introduction of the dehydrating agent toluene will endanger human health, and unreacted boric acid will precipitate when the synthesized BPF dissolves in acetone.

The boric acid ester generated by the above esterification reaction easily absorbs water, and the synthesis process is difficult. The above problem can be solved by using the boric acid ester as a raw material. BPF produced by the reaction of triphenyl borate and paraformaldehyde at 130 °C exhibits a solid form, whereas BPF prepared at 90 °C and 120 °C initially appears as a viscous liquid, which solidifies upon cooling to room temperature and turns into a viscous liquid upon reheating. This behavior demonstrates favorable processability [10].

The yield of BPF synthesized by the paraformaldehyde method is high, the phenolic hydroxyl group of phenol is blocked, and the heat resistance and strength of the resin are not affected by the water absorption and oxidation of the phenolic hydroxyl group. However, the reaction process poses challenges in terms of control, as gelation or explosion can occur [11]. The reaction conditions are harsh, and the quality and stability of the resin are greatly affected by time [12].

### 2.2. Salicyl Alcohol Method

**Figure 2:**
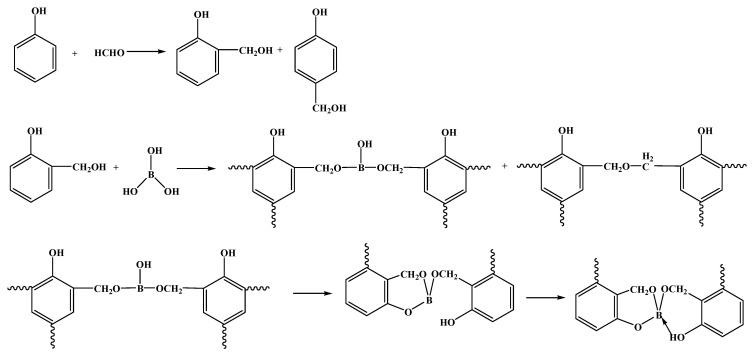
The synthetic route of BPF by salicyl alcohol method [5].

The salicyl alcohol method [5] involves the condensation reaction of phenolic compounds, such as phenol, and formaldehyde solution, in the presence of an alkaline catalyst. This reaction generates salicylic alcohol, which subsequently reacts with boric acid to produce BPF (as shown in Figure 2).

Factors that affect the activity of the reaction system, such as catalyst, aldehyde–phenol–boron ratio, reaction temperature, and time, affect the performance of BPF [13]. Therefore, the exploration of the optimal synthesis conditions for the salicyl alcohol method has received a lot of attention [14,15,16,17]. For instance, when the phenol:formaldehyde:boric acid molar ratio is 1:1.5:0.4, the condensation temperature is 60~70 °C, the boron esterification temperature is 105~120 °C, and ammonia water (pH = 8~9) is used as a catalyst, the residual carbon rate of BPF at 700 °C is 75.3% [14]. In another study, it was shown that when the phenol:formaldehyde:boric acid molar ratio is 1:(1.7~2.0):0.3, the reaction time is 2.0 h, and sodium carbonate is used as a catalyst, the residual carbon rate of BPF at 800 °C is 65.88% [15]. Similarly, with a phenol:formaldehyde:boric acid molar ratio of 1:1.3:0.3 and sodium carbonate as the catalyst, the oxidation temperature of BPF is about 50 °C higher than that of pure phenolic resin and the residual carbon rate at 900 °C is higher than 75% [16]. Moreover, by using a molar ratio of phenol:formaldehyde:boric acid of 1:2:0.3, a condensation reaction temperature of 70~75 °C, a boron esterification reaction temperature of 100~104 °C, one dehydration step, and sodium carbonate as the catalyst, the residual carbon rate of BPF at 900 °C is as high as 76.38% [17]. However, when the molar ratio was 1:1.8:0.45, although the residual carbon rate was the highest, the viscosity in the later stage of the reaction was very high, the phenomenon of gelation often appeared, and the reaction was difficult to control.

**Figure 3:**
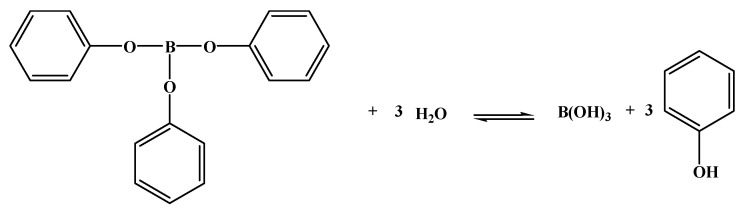
Hydrolysis reaction of BPF [18].

In addition, the influence of the type of raw material phenol [18] and boron [19] on the performance of BPF should not be ignored. Phenol [20,21,22], bisphenol A [23,24], or bisphenol F [25] was reacted with formaldehyde solution under alkaline conditions at 65–70 °C for 1 h by Jungang Gao et al., and the resulting solution was dehydrated under reduced pressure to obtain salicyl alcohol. Subsequently, boric acid was added, and the mixture was reacted slowly at 100~110 °C for 40~60 min and finally dehydrated slowly. This process yielded phenol type, bisphenol A type, and bisphenol F type BPFs. The B-O bond in the phenol type BPF is highly sensitive to humid air, it decomposes in the presence of water, an unstable complex was generated, alcohol was removed, and boric acid was formed (as shown in Figure 3). Because the boron atoms of bisphenol A type and bisphenol F type BPFs are saturated with coordination, the water resistance of bisphenol A type and bisphenol F type BPF is better than that of phenol type BPF [18]. However, the viscosity of bisphenol A type BPF is high, it is difficult to stir in the later stage of the reaction, and the price of the raw material bisphenol A is higher than that of phenol [26]. In addition, the rigid sulfone groups in the main chain of bisphenol S type BPF restrict the rotation of the main chain, which improves the thermal and mechanical properties of the resin [27] and makes the production process difficult [28].

The BPF prepared by the above salicyl alcohol method forms a large amount of yellow precipitate immediately after cooling. Infrared analysis shows that the precipitate primarily consists of boric acid. It may be due to the fact that when the temperature is high, boric acid will dissolve in the solution to form a homogeneous system and, when the temperature is low, the solubility of boric acid decreases and it precipitates out of the solution [10]. When boric acid reacts with benzyl alcohol for 50 min, the reaction conversion rate is more than 50%; when boric acid reacts with phenol for 150 min, the reaction conversion rate is only 4%, and most of the boric acid that did not participate in the reaction will precipitate after the stirring is stopped, indicating that the reaction reactivity of boric acid with phenol is much lower than that of boric acid with benzyl alcohol [20,23]. The unreacted phenolic hydroxyl groups in the synthesis stage first form B-O bonds with the boronic acid molecules in the curing stage, and as the curing temperature increases to 130~200 °C, the boron atoms and oxygen atoms coordinate to form a six-membered ring structure [21,22,25], and the thermal stability and water resistance of BPFs are improved. When the curing temperature continued to increase to above 200 °C, some of the B-O bonds were oxidatively broken [29]. With the increase in boron content, the probability of boron participating in coordination decreases and the six-membered ring structure is more difficult to form in bisphenol F type BPF than in bisphenol A type BPF [25].

**Figure 4:**
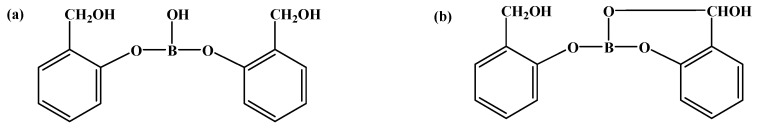
Condensation products from the reaction of salicyl alcohol and boric acid [30]. (**a**) Molecules of salicyl alcohol (**b**) product containing methylol group condensation.

Due to the high conversion rate and high speed of the reaction between boric acid and benzyl alcohol [20], the BPF products synthesized by using salicyl alcohol as raw material to reflux react with boric acid for 4 h are pure, almost all of them are diphenyl borate (Figure 4a), and a small amount of diphenyl borate continues to undergo intramolecular reactions to form a six-membered ring structure (as shown in Figure 4b) [30]. Compared with pure phenolic resin, under nitrogen atmosphere, the weight loss of the cured BPF at 500 °C is 5%, and the residual carbon rate (75%) at 800 °C is much higher than that of pure phenolic resin (45%). In air, the weight loss rate of the cured BPF at 500 °C is 10%, and the residual carbon rate at 800 °C is as high as 72%. Meanwhile, the mechanical properties of BPF are better than those of pure phenolic resin [30].

In the final stage of synthesis of the above-mentioned BPF, the viscous resin is generally poured out while it is still hot. However, this method is difficult to realize in industrial production. In order to facilitate the discharge and direct use in the fields of premix and adhesives, alcohol, ethylene glycol, etc. are usually added to form a resin solution before discharge [31,32,33].

The synthesis process and equipment of the salicylic alcohol method are simple, and the product quality is easy to control. However, the yield is low, and some metal ions introduced with the catalyst will adversely affect the electrical properties and oxidation resistance of the resin [34,35]. In addition, due to the more complete reaction between boric acid and phenol and the higher proportion of boric esters formed, the BPF synthesized by the paraformaldehyde method exhibits a higher thermal decomposition temperature and better hydrolysis resistance than the BPF synthesized by the salicyl alcohol method [36].

### 2.3. Copolymerization and Blending Method

The copolymerization and blending method is a method of synthesizing BPF by adding boride at the end of the synthesis procedure of phenolic resin or during the processing of the blend, so that a portion of the boride can participate in the reaction.

When boric acid was added at the end of the synthesis reaction of thermosetting phenolic resin, the residual carbon rate of the resulting BPF at 800 °C was as high as 70% [37,38]. The introduction of boron into a phenolic resin system can accelerate the curing reaction, lower the peak temperature and activation energy of the curing reaction, and facilitate the curing reaction [39]. However, the curing of thermoplastic BPF with hexamethylenetetramine shows that the introduction of boron increases the activation energy of the curing reaction, reduces the curing temperature and the curing reaction rate, and prolongs the curing time of the resin [40]. Zinc acetate-catalyzed thermoplastic BPF possesses a residual carbon rate of up to 61.4% at 750 °C in an air atmosphere and exhibits excellent thermo-oxidative stability and processing fluidity. As the reaction proceeds, the viscosity of the resin system becomes greater and it is difficult for the boric acid to react completely [41].

The BPF obtained by directly mixing and curing phenolic resin powder and boric acid has the highest heat resistance when the amount of boric acid is 10%, and the impact strength is the highest when the amount of boric acid is 5%. As the amount of boric acid continues to increase, the degree of crosslinking of the resin increases, and the impact strength decreases, even lower than that of pure phenolic resin [42].

**Figure 5:**
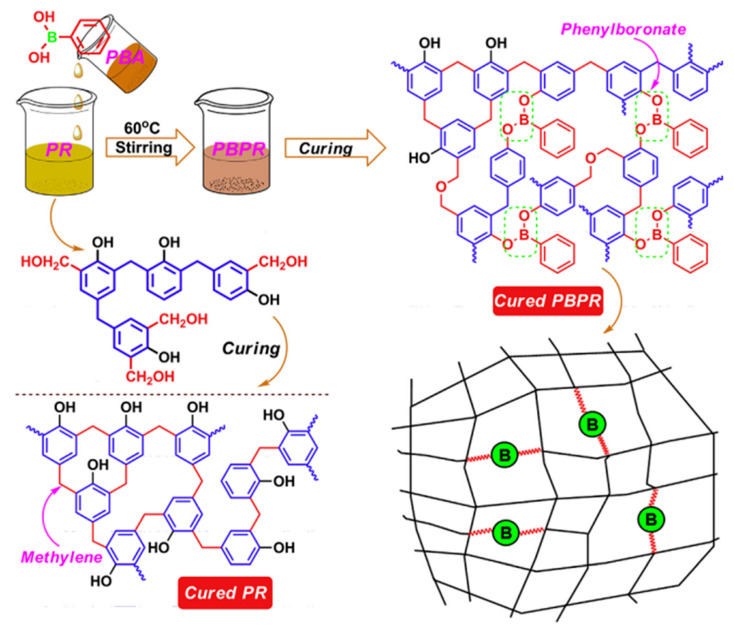
Preparation and structure of the cured PBPR [43].

In order to improve the processability and mechanical properties of the above-mentioned boric acid-modified phenolic resin, Xinli Jing et al. [43,44] prepared phenylboronic acid-modified phenolic resin (PBPR) (Figure 5), which exhibited a much lower viscosity growth rate compared to boric acid-modified phenolic resin [43]. The viscosity of PBPR increased from 10.3 to 31.3 mPa·s at 60 °C for 160 min, while the viscosity of pure phenolic resin increased from 11.4 to 208 mPa·s [44]. The impact strength of the cured PBPR is significantly higher than that of pure phenolic resin [43]. Scanning electron microscopy (SEM) confirmed that the PBPR casting is ductile and the fractured section is scaly, while the pure phenolic resin casting has a smooth fractured section with only a few small holes, showing brittle fracture behavior [44]. Due to the structure, bond energy, reaction activation energy, and steric hindrance effect [4,45], PBPR has excellent thermal stability, and its residual carbon rate (76.2%) at 800 °C is much higher than that of BPF (67.9%) and pure phenolic resin (63.4%) [46].

Due to the challenges posed by the poor solubility and low reactivity of boric acid in organic solvents, hyperbranched borate (HBPB) offers a viable alternative. HBPB is soluble in acetone and has good compatibility with phenolic resins. The hyperbranched borate-modified phenolic resins prepared by solution blending HBPB with phenolic resins can reduce the polymerization temperature and curing rate of phenolic resins [47]. At 800 °C, the residual carbon rates of low-pressure barium phenolic resin, HBPB-modified phenolic resin (PR-HBPB 10) with 10% mass fraction of HBPB, hyperbranched boronate (HBp)-modified phenolic resin (PR-HBp10) with 10% mass fraction of phenolic hydroxyl end groups, and hyperbranched boronate (HBb)-modified phenolic resin (PR-HBb10) with 10% mass fraction of boronic acid hydroxyl end groups were 64.2% [48] (or 63.8% [49]), 71.3% [49,50], 73.7% [47,51], and 75.4% [47,48,51], respectively. The addition of paraformaldehyde (PFM) can improve the curing degree of phenolic resin and further improve its heat resistance. When the added amounts are 0.006 of the mass fractions of HBPB, the residual carbon rates of PR-HBPB10, PR-HBPB50, and PR-HBPB80 can be increased from 71.3%, 62.1%, and 78.4% to 74.9%, 75.4%, and 79.2%, respectively, and its residual carbon rate is the highest among phenolic resins [49]. However, the curing temperatures of PR-HBPB, PR, HBPB, and PFM blend (PR-HBPB-PFM) are too high (up to 220 °C) [49], HBPB, HBp, and HBb are complicated to synthesize and difficult to prepare on a large scale, the hyperbranched molecular weight distributions are wide, and structural controllability is poor [47].

In addition, B_4_C ceramic powder can convert small molecular volatiles such as CO generated by the thermal decomposition of phenolic resin into amorphous carbon and retain them in the resin matrix, which can effectively increase the residual carbon rate and improve the thermal stability of the phenolic resin [52] and improve the high-temperature bonding with Si_3_N_4_ [53] and graphite [54,55]. Although the addition of B_4_C ceramic powder can improve the thermal and oxygen stability of phenolic resin, it will lead to an increase in the viscosity of the resin, an increase in the curing temperature, poor compatibility with the phenolic resin, and poor adhesion between the modified resin and the reinforcing material during the molding process of the composite material, which makes the processing difficult [30,56].

Although the copolymerization and blending method is simple to operate, it does not ensure a complete reaction between the phenolic resin and the boride, affecting the quality and uniformity of the product [57].

In addition to the above three synthetic methods, under certain conditions, phenol or its derivatives, borides, and formaldehyde can also be added simultaneously for copolymerization to prepare BPFs [58,59,60]. This method is easy to operate, but it is difficult to control the degree of intercalation of modified raw materials and the degree of crosslinking of products [61].

In conclusion, in the existing synthesis methods, whether the paraformaldehyde method or salicyl alcohol method, the boron content in BPF is correspondingly low due to the low reactivity of common borides, i.e., it fails to give full play to the role of boron, and most of the BPFs obtained by the preparation process exhibit poor processing properties, which restricts their wide application.

## 3. Modification of BPF

Due to the high viscosity of the BPF in the later stage of synthesis, it tends to gel and is difficult to control and, at the same time, the storage stability of BPF (especially the solid powder resin) is poor and it easily agglomerates. In addition, the B-O bond is easy to hydrolyze, resulting in poor water resistance of BPF [62]. For this reason, it is necessary to further modify the heat resistance, toughness, and water resistance of BPF. The modification of BPF can be divided into three categories: organic modification, inorganic modification, and nanomaterial modification [63].

### 3.1. Organic Modification

#### 3.1.1. Silicone Modification

Silicone modification involves the reaction between the active groups present in silicone monomers and the phenolic hydroxyl, methylol, and boron hydroxyl groups of the BPF can improve the performances of the BPF. The addition of silicone endows BPF with low viscosity and makes it easy to control synthesis [62,64], the surface tension of BPF is significantly reduced, the water resistance and storage stability are improved [62,65,66], and the flexible polysiloxane chain introduced into the modified resin can improve the toughness of BPF [67].

The salicyl alcohol method is generally adopted to synthesize organosilicon-modified BPF. In the later stage of dehydration, silicone modifiers such as organosilicon prepolymer [68], hydroxyl-terminated organosilicon prepolymer [62], silane coupling agent KH550 [65], tetraethyl orthoslicate [67], and tetramethoxysilane [69] are added. The organic silicon prepolymer-modified BPF exhibits a remarkable residual carbon rate of up to 79% at 800 °C in a nitrogen atmosphere after curing, making it suitable for applications in temperature-resistant and anti-ablation coatings or composite materials. However, the modified resin tends to produce insoluble matter when dissolved in ethanol [68]. The residual carbon rate (75%) of the hydroxyl-terminated organosilicon prepolymer-modified BPF at 800 °C in nitrogen atmosphere is about 50% higher than that of ordinary 213 phenolic resin (24.4%), but insoluble matter is produced when the modified resin is dissolved in ethanol and acetone [62]. The residual carbon rate (37.69%) of KH550-modified BPF at 800 °C in air atmosphere is about 15% higher than that of ordinary phenolic resin (22.64%) [65].

The optimum synthesis conditions for common thermosetting phenolic resin were determined by orthogonal experiments. On this basis, BPFs and borosilicate phenolic resins were synthesized using boric acid and phenyltriethoxysilane as raw materials. The gel time and free phenol content of the modified phenolic resin decreased, but the molecular weight increased. The initial decomposition temperatures of common phenolic resin, BPF, and borosilicate phenolic resin are 350 °C, 450 °C, and 400 °C, respectively. Moreover, the residual carbon rates at 800 °C are 52.7%, 69.03%, and 65.00%, respectively [64]. Although the heat resistance of borosilicate phenolic resin is worse than that of common phenolic resin, its heat resistance is not as good as that of BPF, probably because the silicone segment hinders the formation of a three-dimensional network structure during resin curing [62,65]. In order to eliminate the influence of residual reactants on the resin properties, the borosilicate phenolic was purified and separated, and the residual carbon rate at 800 °C was increased to 77.0% [70].

Boric acid was first reacted with organosilicon prepolymer to synthesize polyborosilane and then condensed with phenolic resin by Bin Zhang et al. [71], polyborosiloxane-modified phenolic resin (BSP) was obtained, and the residual carbon rate of BSP at 800 °C was 69%. This research group [67] used another method to prepare boron- and silicone-containing phenolic resin (BSiPF), in which silicone prepolymer and boric acid were introduced in the synthesis process of phenolic resin and SiO_2_ formed by in situ hydrolysis of ethyl orthosilicate was used to further modify BSiPF. Thermogravimetric analysis conducted in an air atmosphere showed that compared with ordinary phenolic resin, the BSiPF with SiO_2_ formed in situ had significantly better heat resistance, its initial decomposition temperature in air atmosphere was 475 °C, and the residual carbon rate at 1000 °C was 21%, but the initial decomposition temperature of ordinary phenolic resin was about 410 °C, and it had close to complete weight loss at 700 °C.

Another approach by Tong Zhao et al. [72] involves dissolving silicon phenolic resin in ethanol, adding boric acid and hexamethylenetetramine, and dehydrating the mixture at room temperature under vacuum to prepare BSiPF. In nitrogen atmosphere, the initial decomposition temperature of BSiPF was as high as 434.5 °C, and its residual carbon rate at 900 °C reached 72.5%, which was 6.2% higher than that of thermoplastic phenolic resin. In air atmosphere, its residual carbon rate at 900 °C was 16.7% higher than that of thermoplastic phenolic resin and the maximum thermal decomposition rate decreased by about 2%/min. This research group [73] synthesized two polyborosiloxanes (PBSis) through a similar method by reacting boric acid with phenyltrimethoxysiloxane directly without any catalyst. By combining PBSi with phenolic resin, several PBSi-modified phenolic resin blends (PBSi/PFs) were prepared. The residual carbon rate of the PBSi/PF cured product with sea-island structure was higher than that of a common phenolic resin. In nitrogen atmosphere at 800 °C, the residual carbon rate of PBSi/PF resin with a mass ratio of PBSi/PF of 0.4:1 was as high as 70.83%, and silicon boron oxycarbide (SiBOC) ceramics were formed by ceramization of PBSi/PFs under this condition. The silicon borocarbonitride (SiBCN) ceramics formed by the pyrolysis of polyborosilazane (PBSZ) can withstand a temperature up to 2000 °C and have excellent temperature resistance. Therefore, PBSZ can be used to modify phenolic resins. With the increase in the mass fraction of PBSZ [74], the thermal decomposition temperature and residual carbon rate at 900 °C of PBSZ-modified phenolic resin (SBPR) both increased. When the mass fraction of PBSZ was higher than 40%, the improvement effect of both was not obvious. In nitrogen atmosphere, the thermal decomposition temperature and residual carbon rate of pure phenolic resin cured with 10% hexamethylenetetramine at 900 °C were 259.7 °C and 58.57%, respectively, which were 138.3 °C and 14.83% lower than those of SBPR with 40% PBSZ mass fraction, respectively. Although SBPR has excellent heat resistance, the phenolic hydroxyl groups in phenolic resin have high reactivity with a large number of B-N bonds in PBSZ, PBSZ was easy to gel when blended with phenolic resin, and SBPR curing was accompanied by the release of large amounts of ammonia gas.

(2-Hydroxyphenyl) propyl silicone oil (Si-phenol) was obtained via Williamson ether synthesis, Claisen rearrangement, and hydrosilylation, and then was mixed with phenol [75]. The silicone-modified boric phenolic resin (SBPF) was synthesized via borate esterifieation. When the content of Si-phenol is moderate (10%), the overall performance of SBPF is the best. Compared with boron phenolic resin, SBPF not only has certain heat resistance, its tensile strength and room temperature shear strength are also improved and especially the impact strength is significantly improved. The residual carbon rate of SBPF at 800 °C (65.2%) was significantly lower than that of BPF (79.5%), which should be due to the introduced flexible chain segments increasing the steric hindrance and reducing the crosslink density of the resin.

**Figure 6:**
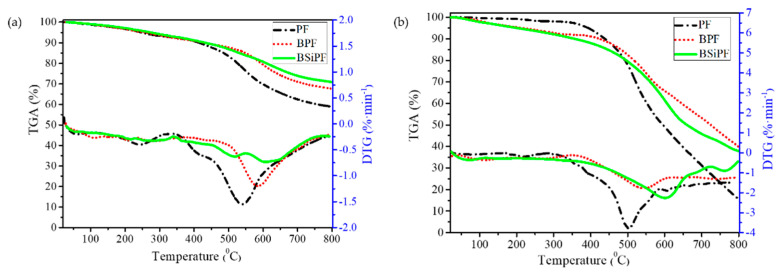
TGA and DTG curves of ordinary PF, BPF, and BSiPF in nitrogen (**a**) and N_2_ (**b**) [76].

In order to improve the thermal stability, oxidation resistance, and storage stability of phenolic resin and to avoid complicated synthetic methods, we designed a facile, environmentally friendly, controllable, and low-cost strategy to prepare a novel boron and BSiPF, in which silicone was reacted with commercial BPF [76]. The as-prepared BSiPF solution has a long shelf life (more than 8 months) at room temperature. In N_2_ and air atmosphere, the residual carbon rates of BSiPF at 800 °C are 70.8% and 37.9%, respectively, which are 12.0% (Figure 6a) and 22.5% (Figure 6b) higher than that of common phenolic resin, demonstrating that silicon and boron have synergic effects in improving the thermal stability and anti-oxidation property of phenolic resin. With superior thermal stability and low viscosity, BSiPF can be used as a novel high-performance matrix resin for ablative materials.

Methyltriethoxysilane [77] was added at the later stage of the synthesis of BPF by the paraformaldehyde method, and the residual carbon rate of the resulting hot-melt BSiPF at 800 °C in a N_2_ atmosphere was as high as 76%. Wang Dong [78] prepared an adhesive by blending hydroquinone-containing polyborosiloxane (PBSP) and titanium-containing titanium borosiloxane (TiBSi) with phenolic resin, respectively. When the mass ratio of PBSP to phenolic resin is 1.5:1, the bonding strength of PBSP/PF is 4.79 MPa, which is slightly higher than that of pure phenolic resin, and the residual carbon rate at 800 °C is as high as 74.10%, which is about 15% higher than that of pure phenolic resin. Similarly, the bonding strength of TiBSi/PF is 7.10 MPa, which is 44%~64.7% higher than that of pure phenolic resin. The residual carbon rate of TiBSi/PF at 800 °C can reach 70.18%.

#### 3.1.2. Amine Modification

The water resistance of BPF is poor due to the unsaturation of the outer nuclear electron layer of boron atoms. For this reason, various amines (such as hexamethylenetetramine [79,80], ammonia water [81,82,83], hexamethylenediamine [84], aniline [84]) were introduced in the synthesis of BPF, through the coordination of nitrogen and boron to form a chelate structure to improve the water resistance of BPF. The water resistance of BPF will be affected by the content of boron and nitrogen and, although increasing the content of boron can improve the heat resistance of the resin, too much boron content will be unfavorable for the formation of B←N coordination bonds and reduce the water resistance of the resin, which limits the application of the resin. Since the possibility of formation of B←N coordination bonds is greater than that of B←O coordination bonds, the higher the N content, the more B←N coordination bonds in the product and the better the water resistance [81].

However, the above-mentioned boron–nitrogen coordination bonds are generally formed in the high-temperature curing stage of the resin [83,84], and hydrolysis will occur during the storage period of the resin, which will affect its performance. The use of hydroxylamine as a modifier to synthesize fully coordinated boron–nitrogen phenolic resin by the paraformaldehyde method can greatly improve the water resistance of BPF [57,85].

#### 3.1.3. Vegetable Oil Modification

The conjugated double bond in vegetable oil can undergo nucleophilic substitution with the ortho and para hydrogens of the phenolic hydroxyl group in the phenolic resin, providing a means for modifying the phenolic resin [86]. The addition of cashew nut oil can improve the hydrolysis resistance [26] and thermal stability [87] of BPF, and the addition of tung oil can improve the flexibility [88] and oil solubility [89] of BPF.

#### 3.1.4. Rubber Modification

Due to the similar solubility parameters, nitrile rubber has good compatibility with phenolic resin [90], and the nitrile group and double bond in nitrile rubber can react with the methylol group in BPF, so nitrile rubber can be used to improve the properties of BPF [91,92]. When the amount of nitrile rubber is 6.7 phr, the impact strength of the modified BPF reaches the maximum value (12.92 KJ·m^−2^), which is 92.8% higher than that of the BPF, and the curing peak temperature of the modified BPF decreases and the heat resistance (below 430 °C) is improved [92].

#### 3.1.5. Bismaleimide Modification

The resin system prepolymer prepared by the copolymerization of bismaleimide resin (BMI) and allyl BPF (XBPF) is soluble in acetone and has good storage performance in a sealed state. With the increase in the allyl content, the heat resistance, mechanical properties, and water resistance of the cured resin are improved. Adding hexamethylenetetramine to XBPF can generate B←N coordination bonds, which can significantly improve the water resistance of XBPF/BMI copolymer resins [93].

#### 3.1.6. Aromatic Hydrocarbon Modification

The thermal decomposition temperature of phenolic resin modified with para-substituted phenol-dominated alkylphenol and boric acid is 449 °C, which is between those of 2123 phenolic resin and bisphenol A type BPF or phenol type BPF [94]. The presence of the para-long chain of alkylphenol improves the flexibility of the resin but makes the resin molecule partially linear rather than bulk, and the reaction rate (relative activity) of the alkylphenol is low, resulting in a decrease in the heat resistance of the resin [95].

### 3.2. Inorganic Modification

#### 3.2.1. Molybdenum Modification

Due to the high bond energy, Mo-O bonds can be introduced to further improve the heat resistance of BPF. Since molybdic acid easily reacts with salicyl alcohol and struggles to undergo esterification with phenol, the synthesis of boron- and molybdenum-modified phenolic resin usually generates methylol phenol first, and then undergoes esterification with ammonium molybdate and boric acid.

Under the premise that the reaction temperature of the first step is 70 °C and the reaction time is 1 h, the residual carbon rate is used as the detection index. The orthogonal test determines the optimal synthesis process of BPF, and the reaction temperature of the second step is 100 °C. The reaction time is 3 h, the amount of boric acid is 7%, and the aldehyde to phenol molar ratio is 1.4. The best synthesis process of boron- and molybdenum-modified phenolic resin is when the reaction temperature of the second step is 105 °C, the reaction time is 2 h, the amount of ammonium molybdate is 4%, the amount of boric acid is 5%, and the molar ratio of aldehyde to phenol is 1.2 [11,94]. After the BPF prepared under the optimum synthesis conditions is heated in a muffle furnace at 800 °C for 7 min, its residual carbon rate is 62.13%, and the thermogravimetric analysis in an air atmosphere shows that its residual carbon rate at 600 °C is 79.7%. The residual carbon rate of the boron- and molybdenum-modified phenolic resin prepared under the optimal synthesis conditions is 62.36%, the residual carbon rate at 600 °C is as high as 85.2%, which is 29% higher than that of pure phenolic resin, and 70 °C increases the initial decomposition temperature. In addition, the free phenol content of the boron- and molybdenum-modified phenolic resin is 3.34% lower than that of the pure phenolic resin, and the impact strength is increased by 120%.

#### 3.2.2. Phosphorus Modification

The addition of phosphoric acid and boric acid can increase the degree of polymerization of the phenolic resin and improve the heat resistance and mechanical properties of the resin. When the amounts of phosphoric acid and boric acid are 1% and 3% of that of phenol, the properties of phosphoric acid–boric acid-modified phenolic resin are the best, the thermal decomposition temperature is 25.9% higher than that of the unmodified resin, reaching 500 °C, and the flexural strength and impact strength are 17.6% and 114.3% higher than those of the unmodified resin, reaching 51.76 MPa and 0.15 J·cm^−2^ [96].

#### 3.2.3. Molybdenum Phosphorus Modification

Taking molecular weight (or degree of polymerization) as the detection index, the determined optimal raw material ratio is when the added amount of ammonium molybdate, phosphoric acid, and boric acid is 1%, 1%, and 5% of the mass of phenol. The resulting resin demonstrates a residual carbon rate of 52.3% at 700 °C, a thermal decomposition temperature of 499 °C, a heat resistance about 25% higher than that of ordinary thermoplastic phenolic resin, a flexural strength of 58.12 MPa, and an impact strength of 0.27 J·cm^−2^ [97].

In a word, BPF was modified by chemical grafting and physical blending to improve its heat resistance, toughness, and water resistance. The methods to improve the heat resistance and toughness are usually contradictory. The introduction of organo-silicon and nanomaterials can improve the heat resistance and toughness of BPF and can be used as important methods to modify BPF. However, ease of aggregation, difficulty in dispersion, and high cost of nanomaterials limit their application in modified BPF. Therefore, the combination of a variety of modification methods to improve the comprehensive properties of BPF will be the research focus of BPF modification in the future.

## 4. BPF-Based Composites

Due to its excellent heat resistance and ablation resistance, good mechanical properties, relatively stable high-temperature friction properties, etc., BPF is often used as the matrix resin of composites and has been used in aerospace ablation resistance materials [98,99,100], friction materials [18,101,102], electrical insulation materials [35,103], and other fields.

### 4.1. Molding Methods

The molding process is the basis for the development of the composites industry. Different molding processes will lead to differences in the structure of the product, which in turn affects the properties of the product [104,105].

#### 4.1.1. Compression Molding

Compression molding involves the uniform mixing of BPF with reinforced fibers, special fillers, or rubber to prepare premix [102,106,107,108,109,110,111]. Alternatively, fiber cloth can be immersed in BPF solution to prepare prepreg [8,33,87,99,100,103,105,112,113]. The prepreg is then dried, placed in a mold, and sealed under heat and pressure to crosslink and solidify the resin. After demolding, a composite product with the same shape as the cavity can be obtained.

#### 4.1.2. Mechanical Mixing Method

In addition to the molding method, the BPF, reinforced fibers, and rubber can also be mechanically mixed first and then vulcanized to prepare composites [114,115,116]. Composites can also be prepared by ball milling BPF, graphene oxide, reinforced fibers, etc., followed by mechanical mixing and pulverization [117].

#### 4.1.3. Bulk Dipping Method

A new type of highly efficient thermal protection material—phenolic-impregnated carbon ablator (PICA)—with low density, low thermal conductivity, and low ablation can be prepared by impregnating the fibrous three-dimensional porous block with BPF solution, drying, and curing [118,119].

#### 4.1.4. Sol–Gel Method

The BPF prepolymer and fir powder were poured into a mold and kept at 80 °C for 24 h, and the reaction system underwent a sol–gel reaction to obtain BPF/wood powder composites [120,121].

#### 4.1.5. In Situ Polymerization

In situ polymerization is a common method for preparing polymer-based nanocomposites. Under the action of a catalyst, phenol, formaldehyde, and nanofillers are reacted to a certain extent and then dehydrated, and boric acid is added to continue the reaction and then dehydrated again to obtain BPF-based nanocomposites [36,122,123,124]. The in situ polymerization method ensures the uniform dispersion of nanofillers in the phenolic resin matrix, enhances the interaction force between the filler and the resin matrix, and is good for stress transfer [125] but increases the viscosity of the reaction system and easily introduces incompletely reacted monomers or impurities, and the end-capping effect of some fillers (such as carbon nanotubes) can shorten the length of the molecular chain of phenolic resin, affecting the polymerization reaction and the strength of composites [126].

#### 4.1.6. Melt Blend Extrusion Method

Different from the molding process of the above-mentioned thermosetting BPF-based composites, thermoplastic BPF-based composite material can be melt-blended, extruded, and pelletized by thermoplastic BPF and flame retardant, such as nylon (or glass fiber) [41,127].

Among the many molding processes reported above, compression molding will remain the most commonly used molding method for BPF-based composites in the future due to its low equipment energy consumption, high production efficiency, easy control of the process window, and high product dimensional accuracy.

### 4.2. Properties

#### 4.2.1. Heat Resistance

Fiber-reinforced phenolic resin-based composites are currently the most important thermal protection materials, and their heat resistance is related to the curing process and fiber content. The residual carbon rate of carbon cloth/BPF after curing in a hydraulic kettle is 80%, and the residual carbon rate of postcured carbon cloth/BPF can reach 87% [98]. The heat resistance of BPF-based composites increased first and then decreased with the increase in glass fiber content. Before the fiber content reached the critical value, the glass fiber increased the steric hindrance of the thermal motion of the BPF polymer segment. After the critical value, the resin content in the composite material was too low, the system was difficult to integrate, and the heat could not be effectively transferred [128].

In addition to being composited with fibers, BPFs can also be in situ polymerized with nanofillers (such as carbon nanotubes, montmorillonite) to prepare composites. Surface modification of nanofillers can improve their dispersibility and their compatibility with BPF, which in turn enhances the heat resistance of the resin. When adding 1 wt% modified carbon nanotubes (m-MWCNTs), the thermal decomposition temperature (T_d_) and residual carbon rate of m-MWCNTs/BPF nanocomposites at 800 °C are as high as 470.7 °C and 72.2%, respectively, which are 36.7 °C and 6.2% higher than those of BPF [124]. Compared with the modified montmorillonite/phenolic resin nanocomposite, the T_d_ and the residual carbon rate at 790 °C of the modified montmorillonite/ BPF nanocomposite can be increased by up to 57 °C and 9.2%, respectively [36]. The SiO_2_/BPF nanocomposites are prepared by in situ polymerization of BPF and ethyl orthosilicate, and the initial thermal decomposition (mass loss of 5%) temperature T_5%_ of the 3wt% SiO_2_/BPF nanocomposite is 487.7 °C, which is 12.4 °C higher than that of BPF; its residual carbon rate at 900 °C is 62.28%, which is 11.23% higher than that of BPF [129]. On this basis, the nano-SiO_2_ hybrid BPF was dissolved in ethanol, mixed with bisphenol A type epoxy resin, impregnated with glass fiber cloth, and then molded to obtain epoxy resin/bisphenol S type BPF/nano-SiO_2_ glass fiber-reinforced composites. As the content of nano-SiO_2_ increases, the T_5%_ of the composite first increases and then decreases. When the mass fraction of nano-SiO_2_ is 3%, the T_5%_ of the composite is the largest, reaching 335.1 °C, 18.3 °C higher than that of the composite without nano-SiO_2_ [39].

When the added amount of GO is 0.5% of the mass of phenol, the residual carbon rate of GO-modified BPF (GO-BPF) nanocomposites at 800 °C is 70.41%, which is significantly better than that of graphene-modified BPF nanocomposites (53.08%) and ordinary BPF (68.31%) [106]. Under N_2_ or air atmosphere, the residual carbon rates of GO-BPFs with different GO additions are higher than that of BPF. Under N_2_, the modified BPF (0.5% GO-BPF) with 0.5% GO has the highest residual carbon rate. When the GO content is more than 0.5%, GO easily agglomerates and struggles to disperse, resulting in the decrease in residual carbon rates of GO-BPFs. However, under air atmosphere, the residual carbon rate of 0.5% GO-BPF is the lowest, which may be due to the complete reaction of 0.5% GO-BPF with oxygen in the air and the high carbon content of the resin [117].

Since ceramizable resin can be decomposed into a ceramic protective layer at a high temperature to protect the inner material, ceramizable resin-based composites can be used as a new type of thermal protection material [130]. THC-400 BPF was used as matrix, microcrystalline mica treated with the coupling agent was used as the filler, and BPF/microcrystalline mica composites were prepared [131,132]. Before the violent thermal decomposition of BPF, flux oxides (Fe_2_O_3_ and MgO) melted and a protective ceramic layer formed, and the ceramic layer and oxidation resistance of microcrystalline mica prevented the oxidative decomposition of BPF, which resulted in a higher thermo-oxidative stability of the BPF/microcrystalline mica composite than BPF. When the additive amount of microcrystalline mica is 5%, the T_d_ and residual carbon rate at 1000 °C of the BPF/microcrystalline mica composite are 113 °C and 4.18% higher than those of the BPF [131].

Different from the above-mentioned fluxes (Fe_2_O_3_ and MgO), B, Bi, and C in the fluxes B_2_O_3_ and Bi_2_O_3_ belong to the adjacent group elements, and their valence structures are similar. At 580~900 °C, the rearrangement and accumulation of the carbon structure can be promoted and the elimination of some groups in MgO-Al_2_O_3_-SiO_2_/BPF (MAS/BPF) ceramic composites is accelerated. A large number of metal ions in fluxes such as Bi_2_O_3_ and glass frit can be used as catalysts for polymer segment cracking to accelerate the cracking of the resin matrix [108]. The ceramic filler nano-Al_2_O_3_ can dissipate heat evenly and absorb a lot of heat after melting; the molten nano-Al_2_O_3_ has high viscosity, strong adhesion to the matrix and reinforced fibers, partially seals the holes, and improves the heat resistance of the composite [87].

#### 4.2.2. Ablative Property

Due to its good ablation resistance, and especially its outstanding instantaneous high temperature ablation resistance and good erosion resistance, phenolic resin has been used as a matrix material for high-temperature- and ablation-resistant composites in the aerospace industry such as spacecraft and missiles for a long time [133]. Liansheng Yan et al. conducted a series of comparative studies on carbon/BPF composites, carbon/polyarylacetylene composites, and carbon/barium phenolic composites [99,100,113,134]. Polyarylacetylene has the best thermal properties, followed by BPF, while barium phenolic resin has the worst, and the residual carbon rate determines the mass retention rate of the matrix after ablation of the composite [134]. Therefore, in terms of mass ablation rate, carbon/polyarylacetylene composites < carbon/BPF composites < carbon/barium phenolic composites. Since the most gas is generated during the pyrolysis of barium phenolic resin, the shear strength of the composite is low and apparent delamination or expansion occurs after ablation, resulting in a decrease in the evident line ablation rate. At the same time, the carbon/BPF sample has a regular ablation profile, and the sample is complete after ablation without delamination. Therefore, in terms of line ablation rate, carbon/polyarylate composites < carbon/barium phenolic composites < carbon/BPF composites. Although the mass ablation rate and line ablation rate of the carbon/polyarylacetylene composite are much smaller than those of the carbon/BPF composite, the former has low molecular polarity and low bonding strength with carbon fibers, resulting in poor mechanical properties of the composite, limiting its application in the aerospace field [113].

Phenylboronic acid-modified phenolic resin (PBPR) is an ideal resin matrix for ablation-resistant materials due to its good thermal stability, manufacturability, and mechanical properties. The ablation resistance of high-silica glass fiber/phenylboronic acid-modified phenolic resin (HSGF/PBPR) composite is significantly better than that of high-silica glass fiber/phenolic resin (HSGF/PR) composites: the linear and mass ablation rates of the latter are 0.180 mm/s and 0.0765 g/s, respectively, while the former’s linear and mass ablation rates are only 0.010 mm/s and 0.0276 g/s [44].

Compared with phenol, resorcinol can provide three more reaction sites when reacting with formaldehyde, and its reactivity is about 12 times that of phenol. Therefore, resorcinol type BPF can be prepared by in situ polymerization under mild reaction conditions [135]. Boric acid and resorcinol form a complex structure at about 120 °C, which improves the heat resistance of BPF. During the ablation decomposition process, the aromatic structure and B-O ester bond in BPF can absorb most of the heat by the heat sink method. At the same time, the formed glass nanosphere thermal protection layer can prevent the penetration of oxygen and further thermal decomposition of the resin. The mass ablation rate and line ablation rate of the BPF composite with 50 wt% boric acid are 0.013 mm/s and 0.048 g/s, respectively, which are 39.30% and 73.40% lower than those of pure phenolic resin. The residual carbon rate at 800 °C is 63%, which is 21% higher than that of pure phenolic resin. When the boric acid content is too high, the viscosity of the system increases sharply, and it easily forms gel or explodes, which is not unfavorable for the reaction [64]. Therefore, after adding formaldehyde, the temperature of the reaction system should not exceed 50 °C [135].

The mass ablation rate of the phenolic-impregnated porous quartz fiber ablation composite is 0.0070 g/s and the line ablation rate is 0.0364 mm/s under the condition of heat flux density of 406.84 kW/m^2^, and the backside temperature gradually increases from the center to the edge of the sample. While under the condition of heat flux density of 659.83 kW/m^2^, the mass ablation rate and line ablation rate of phenolic-impregnated porous carbon fiber ablation composite are 0.0106 g/s and 0.0313 mm/s when ablated for 120 s, and the backside temperature gradually decreases from the center to the edge of the sample [119]. The mass ablation rate and line ablation rate of PICA with a higher density of carbon fiber braid are lower, and the back temperature is lower and vice versa [118]. Table 1 summarizes the ablative properties of fiber-reinforced resin composites [44,99,100,113,134,136,137,138,139,140].

#### 4.2.3. Mechanical Properties

The mechanical properties of BPF-based composites are influenced by the content of glass fiber. The flexural strength and compressive strength of glass fiber-reinforced BPF (GF/BPF) composites increase gradually with the increase in glass fiber content. When the glass fiber content reached 50%, the flexural strength and compressive strength of GF/BPF composites reach the maximum value; the glass fiber content continues to increase, while the flexural strength and compressive strength of GF/BPF composites decrease instead. The impact strength of GF/BPF composites increases with the increase in glass fiber, and it reaches the maximum value when the glass fiber content is 70% [128].

The GO-BPF resin prepared by in situ polymerization was blended with glass fiber, zinc stearate, and hollow microspheres by ball milling, rolling, and pulverizing to obtain GO-BPF-based composites. Compared with BPF-based composites, due to the strong interfacial bonding of GO with phenolic resin, the flexural, tensile, and impact strengths of 0.5% GO-BPF-based composite are increased by 46%, 38%, and 53%, respectively [117].

In order to further improve the mechanical properties of BPF-based composites, surface treatment of inorganic modifiers is an effective way to improve the compatibility and reactivity with BPFs and to improve the interface between the resin matrix and the reinforced material. Carboxylated carbon nanotubes (MWCNTs-COOH), diaminodiphenylmethane-modified carbon nanotubes (MWCNTs-DDM), and borated carbon nanotubes (MWCNTs-Borate) prepared by the covalent grafting method were introduced into BPF by in situ polymerization, respectively. Each of them was then compounded with glass fiber or carbon fiber. The mechanical properties of the modified carbon nanotube-modified BPF composites prepared by hot pressing are significantly improved. When carbon nanotube with a mass fraction of 0.5% is added, the flexural strength and impact strength of the composite are increased by more than 120% and 60%. The borated carbon nanotubes can participate in the reaction in the in situ polymerization, and the improvement in properties is the largest [141]. The modified carbon nanotubes can physically entangle with the resin molecular chain and can also form hydrogen bonds or covalent bonds with the resin molecules through the groups on its surface. Under the action of external force, carbon nanotubes are not easily separated from the matrix, and many microdeformation zones are generated in the matrix and a lot of energy is absorbed, which can not only transmit the external force it bears but also cause the matrix to yield and consume a lot of impact energy, so as to achieve the effect of toughening and strengthening at the same time [141]. Nanoparticle species affect the reactivity and impact strength of BPFs. Nanometer metal oxide TiO_2_ reduces the reactivity of BPF, nanometer non-metal oxide SiO_2_ improves the reactivity of the resin system, while nanometer amphoteric metal oxide Al_2_O_3_ basically has no effect on the reactivity of the resin system [122]. The maximum impact strengths of nano-TiO_2_-, Al_2_O_3_-, and SiO_2_-modified BPF are 231%, 182%, and 202% of that of ordinary phenolic resin, respectively.

The salicyl alcohol method was adopted to synthesize BPF [20,21,22,23,24,25], ethyl orthosilicate and ethanol were added dropwise after the second decompression dehydration of BPF synthesis [129], and the reaction was carried out at 80 °C for 2–3 h. Ethanol and water were removed under reduced pressure to obtain SiO_2_-modified BPF, which was reinforced with glass fiber to prepare a laminate. The impact strength (22.99 kJ/m^2^) and tensile strength (72.08 MPa) of glass fiber/SiO_2_-modified BPF laminates are 39% and 32%, respectively, higher than those of glass fiber/BPF laminates.

In view of the low mechanical properties of the existing laminates, which cannot meet the requirements of aircraft strength and high reliability, nano-SiO_2_ was introduced into boron novolac epoxy resin, and the laminate composites prepared by impregnating high-strength glass cloth have excellent mechanical properties. Bending strength, tensile strength, compressive strength, impact toughness, and interlaminar shear strength are 638 MPa, 616 MPa, 488 MPa, 24.3 J/cm^2^, and 34.2 MPa, respectively. The composite can be used as a high-temperature-resistant electronic and electrical insulating material and a structural material in the aerospace and mechanical industries [103].

In addition to nanofillers graphene, carbon nanotubes, and SiO_2_, rubber can also significantly modify the mechanical properties of phenolic resins. Powder nitrile-butadiene rubber-modified BPF (NBRFB) and TiO_2_ nanoparticle-modified BPF (TiO_2_FB) were synthesized by in situ polymerization and in situ generation, respectively, and then molded with carbon fiber to form carbon fiber/BPF (CF/FB), carbon fiber/powder nitrile rubber-modified BPF (CF/NBRFB), and carbon fiber/TiO_2_ nanoparticle-modified BPF (CF/TiO_2_FB) composites [142]. Since the NBR phase and the resin matrix form an interlocking sea-island structure, when the copolymer is subjected to an external force, the rubber phase will elastically deform and toughen the material, so the mechanical properties are good, and the mechanical properties of the composites are ranked CF/ NBRFB > CF/FB > CF/Ti0_2_FB.

#### 4.2.4. Friction Performance

The development trend of high speed and heavy load of vehicles and mechanical devices has put forward higher and higher requirements for the instantaneous high-temperature resistance and friction performance of friction materials. BPF is suitable as the matrix of high-temperature brake friction materials. The friction properties of BPF-based composite friction materials are related to the content of the resin matrix [102,143].

Nanomodification of BPF helps to improve the stability of friction coefficient of friction materials, reduce wear rate, and improve thermal recession [144,145,146,147]. After adding 1% borated carbon nanotubes, the wear rate of the BPF-based material is reduced by 43.2%, the friction coefficient and wear rate decay rate are only 10.3% and 28.6%, and the friction surface remains intact [144]. Since the modified carbon nanotubes are distributed in the matrix in a crosslinked state and act as a practical component of the transfer film on the surface of the friction material, the adhesion and ploughing wear of the BPF are effectively suppressed, and the friction surface is basically kept intact [144].

In addition, rubber and tung oil can also be used to improve the friction properties of BPF-based composites [61,101,109,148,149].

#### 4.2.5. Flame-Retardant Properties

Since B_2_O_3_ generated when BPF is burned can adhere to the surface of the burning material to form a dense barrier layer, making the material self-extinguishing [10], and there is little smoke, BPF has application prospects in fireproof coatings [150], infrared precision-guided weapons [151], and so on. BPF can significantly improve the condensed phase flame-retardant effect of the flame-retardant (melamine polyphosphate [127], magnesium hydroxide [41]) system and increase the shielding and barrier properties of the formed carbon layer, thereby improving the flame-retardant properties of composites. The flame-retardant grade of BPF/glass cloth laminate is V-0, while that of ordinary phenolic resin/glass cloth laminate is V-1 [152].

#### 4.2.6. Water Resistance

Compared with the above properties of BPF-based composites, there are few research reports on their water resistance. The water absorption of BPF-based composites is related to the content of the resin matrix. The water absorption of hybrid fiber (carbon fiber and steel fiber)-reinforced FB type BPF composites is lower than that of the corresponding benzoxazine-based composites. When the content of FB type BPF increases, the water absorption first decreases and then remains unchanged. When the FB type BPF content is 16%, a significant decrease occurs; a “sea” phase is formed while the content of FB type BPF is more than 18%. The increase in resin content has little effect on the water absorption of composites [153,154]. The BPF prepolymer is bonded to the hydroxyl groups contained in the cellulose or hemicellulose in the wood flour, blocking most of the hydroxyl groups, effectively controlling the shrinkage and swelling of the wood flour and significantly improving the dimensional stability of BPF/wood flour composites. The water absorption rate of BPF/wood flour composites is much lower than that of pure wood flour, and the water absorption rate of composites increases with the increase in wood flour content [121]. In addition, since the catalyst NaOH greatly improves the synthesis efficiency of BPF, the water absorption of the composite decreases.

BPF/montmorillonite nanocomposites are more absorbent than phenolic/montmorillonite nanocomposites: the room-temperature equilibrium water absorption of the former is about 9–14%, while that of the latter is only 3–4%. The water absorption rate (3.8–6.2%) of BPF prepared by first reacting boric acid with phenol was significantly lower than that of BPF prepared by adding boric acid at the end (13.2–14.8%), which proved that the high water absorption BPF/montmorillonite nanocomposite is caused by unreacted or partially reacted boric acid. The prereaction of boric acid with excess phenol can effectively reduce the water absorption of BPF and its composites [36].

Contrary to the above results, after immersion in distilled water at room temperature (20–30 °C) for 24 h, the FB type BPF/glass cloth laminate has very little quality change and electrical property loss, and its water resistance is better than that of ordinary phenolic glass cloth/laminate [152].

All in all, many molding methods can be used to prepare BPF-based composites. Among them, the most widely used one is compression molding. The as-prepared BPF-based composites exhibit excellent heat resistance, ablative property, mechanical properties, and so on. Modification with nanofillers can further improve the above-mentioned properties of BPF-based composites. However, the dispersibility and compatibility of nanofillers with BPF need to be improved, and the interfacial adhesion between BPF and fiber needs to be strengthened.

## 5. Conclusions and Outlook

In conclusion, BPF and its composites possess remarkable heat resistance, ablation resistance, mechanical properties, friction properties, and flame-retardant properties, making them highly suitable for applications in aerospace, weaponry, automotive braking, and fire-retardant materials. However, there are certain challenges associated with BPF and its composites, such as the low activity and precipitation tendency of boric acid, difficulty in controlling the synthesis process of BPF, high viscosity, curing temperature, and pressure, as well as limitations in molding processability and interlaminar shear strength of BPF-based composites.

To address these challenges, various approaches have been proposed. The incorporation of hyperbranched boric acid ester and phenylboronic acid has shown promise in improving the synthesis process of BPF and enhancing its performance. The addition of resorcinol has demonstrated the potential to enhance the reactivity of boric acid and increase its content in the BPF system. The use of silicone as a modifier has proven effective in reducing BPF viscosity, facilitating synthesis control, reducing resin surface tension, and improving water resistance and storage stability.

In view of these, the authors believe that the future research on BPF and its composites should focus on the following aspects: (1) exploring methods to increase the boron content in BPF and optimizing the synthesis process by focusing on reactivity enhancements; (2) investigating new modification methods to further improve the comprehensive properties of BPF; (3) conducting in-depth study on the relationship between the molding process and properties of BPF-based composites to meet the application needs of advanced materials; (4) continuously exploring the properties of BPF and its composites and expanding their application fields. It is believed that with the continuous deepening of research, the application field of BPF and its composites will become more and more extensive.

## Figures and Tables

**Table 1:** Ablative resistance of fiber-reinforced resin composites [44,99,100,113,134,136,137,138,139,140].

Composites	Linear Ablation Rate/mm·s^−1^	Mass Ablation Rate/g·s^−1^	Ref.	Composites	Linear Ablation Rate/mm·s^−1^	Mass Ablation Rate/g·s^−1^	Ref.
C/FB PR	0.038	0.0414	[99]	Basalt/THC-400 PR	0.242	0.1160	[137]
/Barium PR	0.027	0.0465	[99]	Basalt/THC-800 PR	0.227	0.1078	[137]
C/FB PR	0.0284	0.0356	[134]	Basalt/Ammonia PR	0.286	0.1586	[137]
C/Barium PR	0.0152	0.0514	[134]	Basalt/Boron PR	0.0883	0.0691	[138]
C/PAA	0.0014	0.0168	[134]	Basalt/S-157 PR	0.261	0.109	[138]
C/Boron PR	0.029	0.033	[100]	Basalt/HCY PR	0.0834	0.0732	[138]
C/Barium PR	0.036	0.043	[100]	S-2 GF/ Boron PR	0.0843	0.0656	[139]
C/Boron PR	0.053	0.0330	[113]	S-2 GF/ HCY PR	0.0796	0.0687	[139]
C/Barium PR	0.018	0.0436	[113]	PSA/FB PR	0.123	0.0686	[140]
C/PAA	0.012	0.0166	[113]	Phenolic/FB PR	0.042	0.0648	[140]
Quartz/Barium PR	0.092	0.0707	[136]	Phenolic/Benzoxazine	0.058	0.0714	[140]
Quartz/Benzoxazine	0.032	0.0510	[136]	HSGF/PBPR	0.010	0.0276	[44]

## Data Availability

For all relevant data, contact the publishers of the referenced articles.
